# Evaluating risk prediction models for adults with heart failure: A systematic literature review

**DOI:** 10.1371/journal.pone.0224135

**Published:** 2020-01-15

**Authors:** Gian Luca Di Tanna, Heidi Wirtz, Karen L. Burrows, Gary Globe

**Affiliations:** 1 Statistics Division, The George Institute for Global Health, Sydney, Australia; 2 Global Health Economics, Amgen Inc., Thousand Oaks, CA, United States America; 3 Curo Payer Evidence, Envision Pharma Group, Horsham, United Kingdom; Universita degli Studi di Napoli Federico II, ITALY

## Abstract

**Background:**

The ability to predict risk allows healthcare providers to propose which patients might benefit most from certain therapies, and is relevant to payers’ demands to justify clinical and economic value. To understand the robustness of risk prediction models for heart failure (HF), we conducted a systematic literature review to (1) identify HF risk-prediction models, (2) assess statistical approach and extent of validation, (3) identify common variables, and (4) assess risk of bias (ROB).

**Methods:**

Literature databases were searched from March 2013 to May 2018 to identify risk prediction models conducted in an out-of-hospital setting in adults with HF. Distinct risk prediction variables were ranked according to outcomes assessed and incorporation into the studies. ROB was assessed using Prediction model Risk Of Bias ASsessment Tool (PROBAST).

**Results:**

Of 4720 non-duplicated citations, 40 risk-prediction publications were deemed relevant. Within the 40 publications, 58 models assessed 55 (co)primary outcomes, including all-cause mortality (n = 17), cardiovascular death (n = 9), HF hospitalizations (n = 15), and composite endpoints (n = 14). Few publications reported detail on handling missing data (n = 11; 28%). The discriminatory ability for predicting all-cause mortality, cardiovascular death, and composite endpoints was generally better than for HF hospitalization. 105 distinct predictor variables were identified. Predictors included in >5 publications were: N-terminal prohormone brain-natriuretic peptide, creatinine, blood urea nitrogen, systolic blood pressure, sodium, NYHA class, left ventricular ejection fraction, heart rate, and characteristics including male sex, diabetes, age, and BMI. Only 11/58 (19%) models had overall low ROB, based on our application of PROBAST. In total, 26/58 (45%) models discussed internal validation, and 14/58 (24%) external validation.

**Conclusions:**

The majority of the 58 identified risk-prediction models for HF present particular concerns according to ROB assessment, mainly due to lack of validation and calibration. The potential utility of novel approaches such as machine learning tools is yet to be determined.

**Registration number:**

The SLR was registered in Prospero (ID: CRD42018100709).

## Introduction

Heart failure (HF) is a primary cause of death and disability throughout the world [1], and as advancing age is a distinct predictor of in-hospital mortality and complications in HF [2], the prevalence and incidence of HF is predicted to continue to rise as the population ages [1, 3]. Focused research has led to the approval of various therapies for HF management, including angiotensin-converting enzyme inhibitors, angiotensin-receptor blockers, neprilysin inhibitors, beta-blockers, and mineralocorticoid receptor antagonists [4, 5]. With an integrated management strategy, survival rates among patients with HF have improved [3, 6], although outcomes can be highly variable. In parallel with an increasing prevalence and incidence of HF, the economic burden attributable to HF is also predicted to rise [7, 8], particularly given the chronic nature of HF and the high risk of (re)hospitalization [8]. In the United States, increasing efforts have been made to reduce the 30-day readmission rate, and hospitals with a high readmission ratio face substantial financial penalties from the Centers for Medicare & Medicaid Services [9]. It would therefore be of benefit to healthcare providers and payers to be able to stratify patients based on risk of future outcomes, to optimize treatment strategies across patients with different needs. This affords the opportunity to propose which HF patients might benefit most from given therapies, while also responding to the payers’ demands for clinical and economic value.

A number of risk prediction models have been published to statistically predict the risk of future outcomes associated with HF. Despite these models, clinicians seem reluctant to adopt them in daily practice [10], possibly due to their reliability at the patient level being poor, the variety of approaches to choose from, and/or the complexity of statistical methodologies [11]. Clinicians are aware that HF increases a patient’s cardiovascular (CV) risk, and this complexity may mean that clinicians are reluctant to employ a risk-specific model when they see all patients as high risk. As such, risk prediction models are more likely useful for informing healthcare systems to look for at-risk patients and follow-up to improve outcomes. A number of authors have reviewed available risk prediction models, in an attempt to guide and inform healthcare providers and payers of their relative merits [11–15]. For example, Rahimi et al. concluded that several of the models were well-validated and had clinical value, but also that models varied, particularly with regards to their statistical approach, sample size, population characteristics, and parameters employed for model development [12]. As such, no one model could be clearly recommended.

Via a systematic review of the literature (SLR), we sought to identify and quality assess published risk prediction models for HF. Our aim was to understand the methodological development and validation of relevant models, in order to assess the current landscape and, moreover, to inform subsequent efforts in the development of risk prediction tools for HF. The most commonly reported risk predictors were also investigated, and discrimination and calibration of the models analyzed. As potential for bias is a consideration in risk prediction, each identified model was assessed according to the Prediction model Risk Of Bias ASsessment Tool (PROBAST) [16, 17].

## Materials and methods

### Data sources

MEDLINE, including MEDLINE in progress, EMBASE, and the Cochrane Library Database, including the National Health Service Economic Evaluation Database and the Health Technology Assessment Database, were searched using a combination of search terms (S1 Appendix). Principle and practical guidelines advocated by the Cochrane Collaboration Handbook and the Centre for Reviews and Dissemination were employed (where relevant). The SLR incorporated a standardized methodical and transparent approach that adhered to the Preferred Reporting Items for Systematic Reviews and Meta-Analyses (PRISMA) and Cochrane Collaboration guidelines. The SLR was registered in Prospero (ID: CRD42018100709 see: https://www.crd.york.ac.uk/prospero/display_record.php?RecordID=100709).

### Study eligibility

English-language studies published March 1, 2013 to May 29, 2018 were retained for further review if they involved adult patients with HF, aged ≥18 years, were conducted in an out-of-hospital setting, and documented multivariable models that predicted single- or multiple-HF outcomes in the target population, according to the search strategy (S1 Appendix). Preclinical, pharmacokinetic, or pharmacodynamic studies were excluded. Studies were not eligible for inclusion if they: used clinical outcomes that were considered in-hospital; focused on individual predictions or markers of risk (i.e., non-univariable as this type of model tends to report overly optimistic findings [18]); were a letter, opinion piece, or review article; or used a dataset that did not reflect current clinical practices.

### Study selection

**Fig 1:**
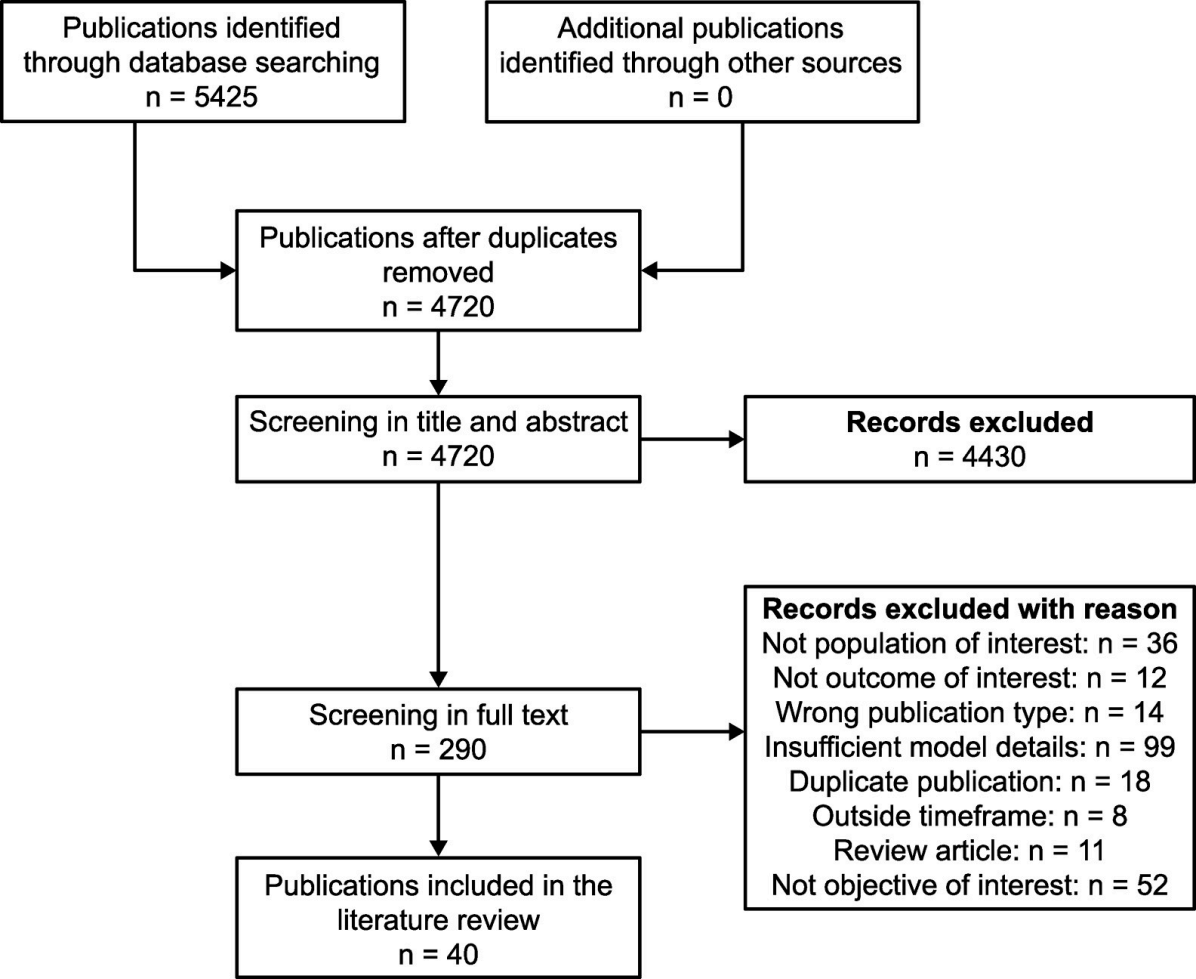
PRISMA flow diagram. PRISMA, Preferred Reporting Items for Systematic Reviews and Meta-Analyses.

Titles and abstracts of identified publications were screened and relevant publications retained for full-text review, according to National Institute for Health and Care Excellence guidance [19] (Fig 1). Both search and screening phases were independently conducted by two trained investigators. Any disagreements were resolved with a senior investigator.

### Data extraction

For each relevant publication, the following information was extracted: study and patient characteristics, candidate variables considered for derivation of the model, final model variables and their association with the outcome, analytical methods, model discrimination, calibration, and validation. Extracted data were examined to identify (but were not limited to): most commonly used candidate predictor variables, type of model and approach used to assess risk predictors, and model performance (e.g., regression approaches, measures of discrimination, calibration, reclassification, and validation), along with their clinical utility among patients with HF. Discriminatory ability was assessed according to standard techniques [20]. Low discriminatory ability was considered as C-statistic <0.60, moderate ability as C-statistic ≥0.60 –<0.70, and good discriminatory ability as C-statistic ≥0.70 [20].

### Analysis of bias (PROBAST)

PROBAST was used to assess the risk of bias (ROB) of each risk prediction model identified from the relevant publications, according to our interpretation of Moons et al. [16]. Each model was assessed for applicability concerns and ROB, according to 3 or 4 domains, respectively. According to guidance from Moons et al. [16], if ≥1 domain is considered “No [N]” or “Probably No [PN]”, there is concern for applicability or potential for bias within that domain. If the review questions were considered to be a good match to the study, concern regarding applicability was rated overall “low” [16]. A publication needed to score “low ROB” in each of the 4 domains for an overall judgment of “low ROB”. However, if ≥1 domain was “high ROB”, a judgment can still be made that the study is overall “low ROB”, but specific reasons should be provided as to why the risk can be considered low [16].

## Results

### Study selection

The SLR yielded 5425 citations, of which 4720 were non-duplicated citations and were further screened. Of these, 290 were retained for full-text review, which led to 40 relevant publications [21–60] (Fig 1). The 250 excluded publications are detailed in Fig 1, with reasons for exclusion.

### Study characteristics

Sample size varied from 43 to 33,349 patients. Patients were aged 59–81 years, and 28–84% of cohorts were male (Table 1). Study follow-up varied considerably (30 days to 5 years). Approximately half of selected publications (n = 17 [46%]) originated from Europe, one-third (n = 10 [27%]) from the United States, 4 from the Asia-Pacific region (11%), and 6 were multinational (16%). The most common comorbidity was type 2 diabetes mellitus (T2DM) (83% [n = 33 studies]), with prevalence of 17–57% across publications. Hypertension was also frequently reported as a comorbidity (n = 28 studies [70%]), with prevalence of 12–87%.

**Table 1:** Characteristics of the patient populations included in the 40 retrieved publications on models of HF risk prediction.

Characteristic	Studies Reporting Characteristic, n (%)	Category	N (%) or [Range]
Age, years	38 (95.0)	---	[59.0–81.3]
Age ≥65 years	4 (10.0)	---	[45.1–86.2]
Male sex	37 (92.5)	---	[28.0–84.0]
Race	12 (30.0)	Caucasian	10 (83.3)
		Black	0 (0)
		Asian	2 (16.7)
		Hispanic	0 (0)
		Other	0 (0)
Sample size	40 (100)	---	[49–33,349]
Study type	40 (100)	Longitudinal	23 (57.5)
		Cross-sectional	0 (0)
		Experimental	5 (12.5)
		Quasi-experimental	0 (0)
		Retrospective	12 (30.0)
Study duration, years	30 (75.0)	---	[30 days–5 years]
Study region	37 (92.5)	Europe	17 (45.9)
		Africa	0 (0)
		North America	10 (27.1)
		South America	0 (0)
		Asia Pacific	4 (10.8)
		Global	6 (16.2)
Current smoker	9 (22.5)	---	[9–33]
Dyslipidemia	11 (27.5)	---	[6.7–74.7]
T2DM	33 (82.5)	---	[17.2–56.5]
Hypertension	28 (70.0)	---	[12–87]
MI	14 (35.0)	---	[17–63]
PAD	9 (22.5)	---	[6.1–16.2]
COPD	20 (50.0)	---	[2.0–28.3]
Atrial fibrillation	24 (60.0)	---	[8.0–63.1]
HF type	26 (65.0)	Chronic HF	15 (57.7)
		Acute HF	9 (34.6)
		Other	2 (7.7)
HF subtype	19 (47.5)	Congestive HF	2 (10.5)
		Acute decompensated HF	7 (36.8)
		HFrEF	5 (26.3)
		HFpEF	1 (5.3)
		Left-sided HF	1 (5.3)
		Right-sided HF	0 (0)
		Other	3 (15.8)

COPD, chronic obstructive pulmonary disease; HF, heart failure; HFpEF, heart failure with preserved ejection fraction; HFrEF, heart failure with reduced ejection fraction; MI, myocardial infarction; PAD, peripheral arterial disease; T2DM, type 2 diabetes mellitus.

Only 26 of retrieved publications specified HF type (65%). The majority of these publications evaluated chronic HF (n = 15/26 [58%]), 9 evaluated acute HF (35%), and the remainder were classified as “other” (2/26 [8%]) (Table 1). Nineteen studies documented HF subtypes; of these 5 reported data specific for reduced ejection fraction (EF) and just 1 for preserved EF.

### Characteristics of risk prediction studies

Nearly half of studies (n = 18 [45%]) failed to provide any indication of data collection period. Of studies that did report study period, data were collected from 2001 to 2015. Few studies reported detail regarding how missing data were handled (n = 11 [28%]); the most common approach being multiple imputation procedures (n = 6 [55%]) (Table 2). Of the 14 studies that reported missing data (35%), the percentage of complete cases ranged between 86% and 100%. Thirty-nine studies (98%) evaluated candidate predictors during model development. Cox regression was used by approximately half of studies (n = 22 [55%]). As would be expected, hazard ratios (n = 25 [64%]) and odds ratios (n = 12 [31%]) were most often used for estimating risk. All publications employed discrimination methods to assess prognostic utility of their model(s). Area under the curve-receiver operating characteristic (AUC-ROC) (n = 19 [48%]) and C-statistic (n = 18 [45%]) were most often used (Table 2).

**Table 2:** Methods reported for model development and validation in the 40 retrieved publications on models of HF risk prediction.

Characteristic	Studies Reporting Characteristic, n (%)	Category	N (%) or [Range]
Missing data reported	14 (35.0)	---	---
Methods for handling missing data	11 (27.5)	Multiple imputation	6 (54.5)
		Nearest neighbor approach	0 (0)
		Complete case analysis	1 (9.1)
		Corresponding median value	1 (9.1)
		Other	3 (27.3)
Percentage of complete cases	10 (25.0)	---	[85.8–100]
Evaluation of candidate predictors	39 (97.5)	Binary logistic regression	1 (2.6)
		Multivariable logistic regression	8 (20.5)
		Mutually adjusted logistic regression	1 (2.6)
		Stepwise logistic regression	1 (2.6)
		Multivariable Cox regression	12 (30.8)
		Stepwise Cox regression	9 (23.1)
		Reduced Cox regression	1 (2.6)
		Machine learning algorithm*	2 (5.1)
		Purposeful variable selection	1 (2.6)
		Other	3 (7.6)
		Odds ratio	12 (30.8)
Measure of risk estimate reported	39 (97.5)	Hazard ratio	25 (64.1)
		Relative risk	0 (0)
		Incidence	0 (0)
		Other	2 (5.1)
		C-statistic	18 (45.0)
Method of discrimination assessed	40 (100)	AUC-ROC	19 (47.5)
		Kaplan-Meier	2 (5.0)
		Concordance index for survival^†^	1 (2.5)
		Hosmer-Lemeshow goodness-of-fit	5 (31.3)
Method of calibration assessed	16 (40.0)	Fine-Gray	2 (12.5)
		Greenwood-Nam-D’Agostino	1 (6.25)
		Gronnesby and Borgan	2 (12.5)
		Pseudo R^2^	1 (6.25)
		Observed and predicted correlation	3 (18.7)
		Other	2 (12.5)
		---	---
NRI assessed	14 (35.0)	---	---
IDI Assessed	6 (15.0)	Bootstrapping	14 (73.7)
Internal validation assessed	19 (47.5)	Cross-validation	2 (10.5)
		Split population	3 (15.8)
		External cohort comparison	8 (88.9)
External validation assessed	9 (22.5)	Other	1 (11.1)
		Other	1 (11.1)

*Machine learning algorithms employed either a Naïve Bates Model, or a Random Forest approach.

^†^The method of Therneau was used to determine the concordance index for predicting survival. AUC-ROC, area under the curve-receiver operating characteristic curve; NRI, net reclassification improvement; IDI, integrated discrimination index.

Beyond model discrimination, steps for evaluating model performance were suboptimal. Less than half of retrieved publications evaluated model fit through calibration methods (n = 16 [40%]). Approaches to correctly classify patients according to severity of HF risk were not widely reported, with net reclassification improvement (NRI) (n = 14 [35%]) or integrated discrimination index (IDI) (n = 6 [15%]) used by a minority of studies (Table 2). Interpretation of these observations is hampered by lack of similarity in approach, particularly as some studies utilized category-dependent NRIs, whereas others a category-free NRI technique. Only 20 studies performed an estimation of internal model validation (50%), with bootstrapping most commonly used. External validation was less frequently reported (n = 10/40 [25%]), with the majority of these publications (n = 8/10 [80%]) employing an external model cohort for comparison (Table 2).

### Risk prediction model outcomes

Within the 40 studies, 55 (co)primary outcomes were assessed, including all-cause mortality (n = 17), CV death (n = 9), HF hospitalizations (n = 15), and composite endpoints (n = 14) (Table 3). Across the 53 outcomes that reported discriminatory values (2 did not), only 1 had “low” discriminatory ability based on a C-statistic of 0.59 (HF hospitalization) [35], the majority were considered “good” (C-statistic ≥0.70; n = 31) or “moderate” (C-statistic ≥0.60 –<0.70; n = 31), as discussed in detail below.

**Table 3:** Predictive performance of 55 model outcomes from the 40 retrieved publications on risk prediction in HF.

First Author (Year)	Data Collection Period	Primary Outcome Assessed	No. of Candidate Predictors	No. of Retained Predictors	Base Model C-Statistic	Predictive Model C-Statistic	Incremental C-Statistic*	Calibration Assessed^†^	NRI Value^‡^	IDI Value
***All-cause mortality***										
Barlera S (2013) **[24]**	2002–2005	All-cause mortality	25	14	0.693	0.700	0.007	Yes	0.048	NR
Behnes M (2016) **[25]**	NR	All-cause mortality	3	1	0.826	0.835	0.009	Yes	0.335	0.027
Bjurman C (2015) **[28]**	2010	All-cause mortality	3	3	NR	0.740	NA	No	0.560	NR
Cabassi A (2013) **[29]**	NR	All-cause mortality	13	1	NR	0.702	NA	Yes	0.089	0.036
Carluccio E (2013) **[30]**	NR	All-cause mortality	14	5	0.740	0.810	0.07	No	0.630	0.087
Carrasco-Sanchez FJ (2014) **[31]**	2009–2010	All-cause mortality	2	1	NR	0.770	NA	Yes	NR	NR
Demissei BG (2016) **[34]**	NR	All-cause mortality	48	6	0.750	0.840	0.09	No	0.860	NR
Eapen ZJ (2013) **[35]**	2005–2009	All-cause mortality	NR	12	NR	0.750	NA	Yes	NR	NR
Ford I (2015)^§^ **[37]**	NR	All-cause mortality	41	10	0.677	0.682	0.005	No	NR	NR
Freudenberger RS (2016)^§^ **[39]**	2002–2010	All-cause mortality	30	8	NR	0.655	NA	No	NR	NR
Jackson CE (2016) **[43]**	2007–2009	All-cause mortality	9	6	0.721	0.730	0.009	No	0.330	NR
Jin M (2017) **[44]**	2012–2015	All-cause mortality	10	3	NR	0.699	NA	No	NR	NR
Keteyian SJ (2016) **[45]**	NR	All-cause mortality	10	4	NR	0.690	NA	No	NR	NR
Lassus J (2013) **[47]**	NR	All-cause mortality	13	2	NR	0.730	NA	No	0.203	0.08
Lenzi J (2016) **[48]**	2011–2012	All-cause mortality	4	4	0.730	0.840	0.11	Yes	NR	NR
Nymo SH (2017)^§^ **[53]**	NR	All-cause mortality	6	6	0.747	0.754	0.007	Yes	0.65	NR
Uszko-Lencer N (2017) **[58]**	NR	All-cause mortality	8	8	NR	0.736	NA	No	NR	NR
***CV mortality***										
Adabag S (2014) **[21]**	NR	CVD mortality	18	6	NR	0.750	NA	Yes	NR	NR
Ahmad T (2014)^§^ **[22]**	NR	PFD	3	3	0.820	0.890	0.07	Yes	NR	NR
Ahmad T (2014)^§^ **[22]**	NR	SCD	3	3	0.680	0.750	0.07	Yes	0.110	NR
Bjurman C (2015)^§^ **[28]**	2010	CVD mortality	3	3	NR	0.680	NA	No	0.450	NR
Ford I (2015)^§^ **[37]**	NR	CVD mortality	41	10	0.683	0.690	0.007	No	NR	NR
Masson S (2018)^§^ **[50]**	NR	CVD mortality	1	1	NR	NR	NA	Yes	0.141	NR
Nymo SH (2017)^§^ **[53]**	NR	CVD mortality	6	6	0.756	0.761	0.005	Yes	0.65	NR
Ramirez J (2017)^§^ **[54]**	2003–2004	SCD	12	6	0.720	0.770	0.05	No	NR	NR
Ramirez J (2017)^§^ **[54]**	2003–2004	PFD	10	4	0.750	0.760	0.01	No	NR	NR
***HF hospitalization***										
Álvarez-García J (2015) **[23]**	2007–2011	HF hospitalization	44	3	NR	0.720	NA	Yes	NR	NR
Behnes M (2016)^§^ **[25]**	NR	HF hospitalization	3	1	0.766	0.777	0.011	Yes	0.223	0.009
Betihavas V (2015) **[26]**	NR	HF hospitalization	27	6	NR	0.800	NA	No	NR	NR
Cubbon RM (2014) **[32]**	2006–2009	HF hospitalization	13	6	NR	0.770	NA	Yes	NR	NR
Fleming LM (2014) **[36]**	2007–2011	HF hospitalization	25	8	NR	0.690	NA	Yes	NR	NR
Formiga F (2017) **[38]**	2013–2014	HF hospitalization	18	18	NR	0.649	NA	No	NR	NR
Frigola-Capell E (2013) **[40]**	2005–2007	HF hospitalization	6	4	NR	0.627	NA	Yes	NR	NR
Eapen ZJ (2013)^§^ **[35]**	2005–2009	HF hospitalization	NR	12	NR	0.590	NA	Yes	NR	NR
Ford I (2015)^§^ **[37]**	NR	HF hospitalization	41	12	0.695	0.702	0.007	No	NR	NR
Krumholz HM (2016) **[46]**	NR	HF hospitalization	110	3	NR	0.650	NA	No	NR	NR
Leong KT (2017) **[49]**	2010–2012	HF hospitalization	27	7	NR	0.760	NA	No	NR	NR
Masson S (2018)^§^ **[50]**	NR	HF hospitalization	1	1	NR	NR	NA	Yes	0.205	NR
Shameer K (2017) **[55]**	2014	HF hospitalization	4205	105	NR	0.780	NA	No	NR	NR
Sudhakar S (2015) **[56]**	2011–2013	HF hospitalization	19	19	NR	0.610	NA	No	NR	NR
Zai AH (2013) **[60]**	2008–2011	HF hospitalization	10	10	NR	0.637	NA	No	NR	NR
***Composite endpoint***										
Bhandari SS (2016) **[27]**	2006–2011	Composite endpoint	2	2	0.670	0.680	0.01	No	0.254	NR
Demissei BG (2017) **[33]**	NR	Composite endpoint	47	17	0.618	0.634	0.016	No	NR	NR
Demissei BG (2016)^§^ **[34]**	NR	Composite endpoint	48	6	0.630	0.680	0.05	No	0.400	NR
Eapen ZJ (2013)^§^ **[35]**	2005–2009	Composite endpoint	NR	10	NR	0.620	NA	Yes	NR	NR
Ford I (2015)^§^ **[37]**	NR	Composite endpoint	41	12	0.676	0.683	0.007	No	NR	NR
Freudenberger RS (2016)^§^ **[39]**	2002–2010	Composite endpoint	NR	NR	NR	0.660	NA	No	NR	NR
Hummel SL (2013) **[41]**	NR	Composite endpoint	13	6	NR	0.716	NA	No	NR	NR
Huynh QL (2016) **[42]**	2014–2015	Composite endpoint	3	3	0.760	0.830	0.07	No	0.174	0.077
Meijers WC (2015) **[51]**	NR	Composite endpoint	29	1	0.712	0.745	0.033	No	–0.048	0.011
Montero-Perez-Barquero M (2015) **[52]**	2008–2013	Composite endpoint	8	8	NR	NR	NA	Yes	NR	NR
Nymo SH (2017) **[53]**	NR	Composite endpoint	6	6	0.728	0.736	0.008	Yes	0.65	NR
Upshaw JN (2016) **[57]**	2001–2005	Composite endpoint	12	12	NR	0.720	NA	Yes	NR	NR
Vader JM (2016) **[59]**	NR	Composite endpoint	NR	9	NR	0.670	NA	No	NR	NR
Vader JM (2016)^§^ **[59]**	NR	Composite endpoint	NR	10	NR	0.690	NA	No	NR	NR

*Difference between base model and predictive model reported.

^†^Calibration values not shown due to heterogeneous types of calibration models used.

^‡^Both categorical and continuous reclassification values are displayed according to publication.

^§^Co-primary endpoints reported.

CVD, cardiovascular disease; HF, heart failure; IDI, integrated discrimination index; NA, not applicable; NR, not reported; NRI, net reclassification improvement; PFD, pump failure death; SCD, sudden cardiac death.

#### All-cause mortality

Of the 17 model outcomes that predicted all-cause mortality, 3 assessed CV mortality as a co-primary endpoint, 3 HF hospitalization, and 5 composite outcomes (Table 3). The median [range] of final candidate variables entered for selection during model development was 10 [2–48], and following candidate variable selection through multivariable modeling, 5 [1–14] variables were retained.

Discriminatory value was assessed for all 17 all-cause mortality outcomes, based on C-statistic (n = 10) or reported as AUC-ROC (n = 7). Relevant model outcomes showed predictive C-statistic values considered “moderate” or “good”, ranging between 0.655 and 0.840 (Table 3). Eight model outcomes provided C-statistics according to a base model in an effort to determine the incremental value when retaining candidate variables into the final model, these C-statistics ranged between 0.677 and 0.826. Internal validation was carried out by 8 model outcomes (47%), primarily by bootstrapping. Just 3 (18%) performed external validation.

#### CV mortality

Of the 9 model outcomes that predicted CV mortality, including sudden cardiac death and pump failure, 3 also modeled all-cause mortality and 1 HF hospitalization. The median [range] of final candidate variables was 6 [1–41], with 4 [1–10] retained within the final model, similar to the number retained for all-cause mortality (Table 3). Of the 9 CV mortality model outcomes, 3 reported C-statistic for model discrimination, 3 reported AUC-ROC, 1 used Kaplan-Meier assessment, and 2 used the Therneau’s survival concordance index. The 8 relevant model outcomes displayed “moderate” or “good” discriminatory values, with a model C-statistic ranging between 0.680 and 0.890 (Table 3). Only 1 model outcomes performed internal validation [53] and none external validation.

#### HF hospitalization

Admission to hospital for HF was the most common endpoint, assessed in 15 models. Of the overall outcomes, 3 additionally assessed all-cause mortality and 1 CV mortality. The median [range] of candidate variables for HF hospitalization was the highest of the 4 outcome categories (19 [1–4205]), although the median number of retained variables was equivalent to those retained for composite endpoints (7 [1–105]). Discrimination was most commonly assessed using the C-statistic (n = 7) or reported as AUC-ROC (n = 7), with 1 model outcomes using Kaplan-Meier assessment. C-statistics ranged between 0.59 and 0.80 (Table 3). Eapen et al. had the largest sample size (33,349 subjects), and a “low” discriminatory value of 0.59 for HF hospitalization [35]. This study assessed all-cause mortality and composite endpoints using different models, and reported good (0.75) and modest (0.62) discrimination, respectively [35] (Table 3). The majority of the predictive model outcomes for HF hospitalization were unable to determine incremental values, as only 2 included a base model. Seven model outcomes (47%) included an assessment of internal validation; 3 (20%) discussed external validation.

#### Composite endpoints

Fourteen model outcomes assessed composite outcomes, with median [range] candidate variables of 7 [1–48], of which the same median number (7 [1–17]) were retained in the final model. The majority of model outcomes (n = 13/14) reported methods of discrimination, most commonly using C-statistic (n = 7) or reporting as AUC-ROC (n = 5); 1 used Kaplan-Meier assessment. When composite outcomes were the endpoint, applicable models displayed “moderate” or “good” discriminatory ability, with C-statistics ranging between 0.620 and 0.745 (Table 3). Six studies (6/13 [46%]) did not report a base model to allow calculation of incremental C-statistic. Eight models (57%) included an assessment of internal validation. Six model outcomes (43%) employed external validation, which was the highest proportion of all 4 outcome categories.

### Model predictors

**Fig 2:**
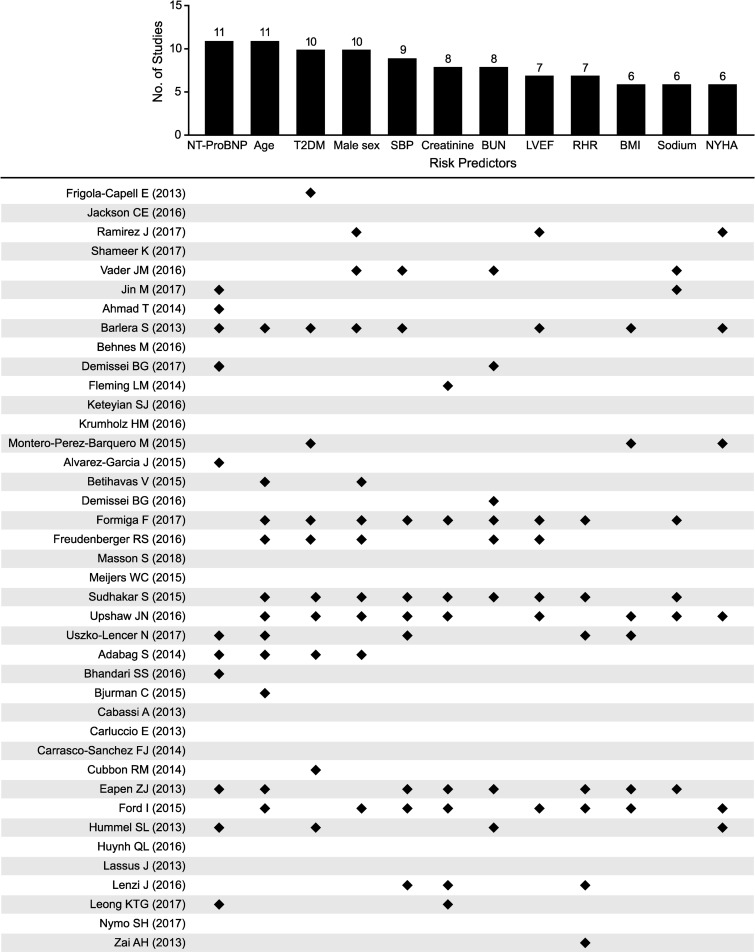
Most common predictors examined in the 40 retrieved publications on models of HF risk prediction. BMI, body mass index; BUN, blood urea nitrogen; HF, heart failure; LVEF, left ventricular ejection fraction; NT-ProBNP, N-terminal prohormone brain natriuretic peptide; NYHA, New York Heart Association; RHR, resting heart rate; SBP, systolic blood pressure; T2DM, type 2 diabetes mellitus.

From the 38 retrieved publications that did not employ machine learning, 105 distinct predictor variables were identified. The 12 most commonly used variables (in >5 publications) were derived from pathophysiological pathways linked to poor health in HF (Fig 2). These included surrogates of demographic, anthropometric, clinical, and laboratory measures. N-terminal prohormone brain natriuretic peptide (NT-proBNP) and age were most commonly included (n = 11 studies each), followed by T2DM and male sex (n = 10 studies each), systolic blood pressure (SBP) (n = 9 studies), blood urine nitrogen (BUN) and creatinine (n = 8 studies each), heart rate and left ventricular EF (n = 7 studies), sodium, body mass index (BMI), and New York Heart Association (NYHA) class (n = 6 studies each) (Fig 2).

Shameer et al. [55] and Krumholz et al. [46] used machine learning and included 4205 and 105 candidate variables, respectively. Despite these large numbers of variables, they did not consider the commonly identified distinct predictors, given in Fig 2. Shameer et al. displayed “good” discriminatory ability with C-statistic of 0.77 [55], suggesting this approach might be promising for predicting relevant outcomes. Conversely, Krumholz et al. documented that a number of socioeconomic, health status, adherence, and psychosocial indicators were not dominant factors for predicting 30-day readmission risk, and model discrimination remained “modest” (C-statistic = 0.65) [46].

### Identification of HF subgroups

Five studies (13%) looked to classify a “high-risk” patient subset. The groups were typically defined according to the highest scoring category, based on each of the included publication’s risk scoring. Álvarez-García et al. [23] demonstrated that patients who presented with 20–30 points on the Redin-SCORE, had a 5-fold increase (i.e., 5.9% vs. 0.9%) in the cumulative incidence of 30-day HF readmission vs. patients scoring 0–19 points [23]. Uszko-Lencer et al. [58] reported 2-year survival probability among patients classified with “high scores” (i.e., BARDICHE-score >16 points) was 58% vs. 97% in the low BARDICHE-score group (≤8 points). Using the Echo Heart Failure Score, Carluccio and colleagues [30] reported that all-cause mortality increased progressively with higher scores (0–5 points). Notably, patients with a score of 5 had an all-cause mortality HR 13.6 points higher than if they had a score of 0. When evaluating “high-risk” on the Heart Failure Patient Severity Index (i.e., decile 10), Hummel et al. [41] noted a 57% increase in 6-month all-cause death and hospitalization (composite), vs. an 8% increase in 6-month combined event rate for those classified as “low-risk” (deciles 1–4).

### PROBAST

**Fig 3:**
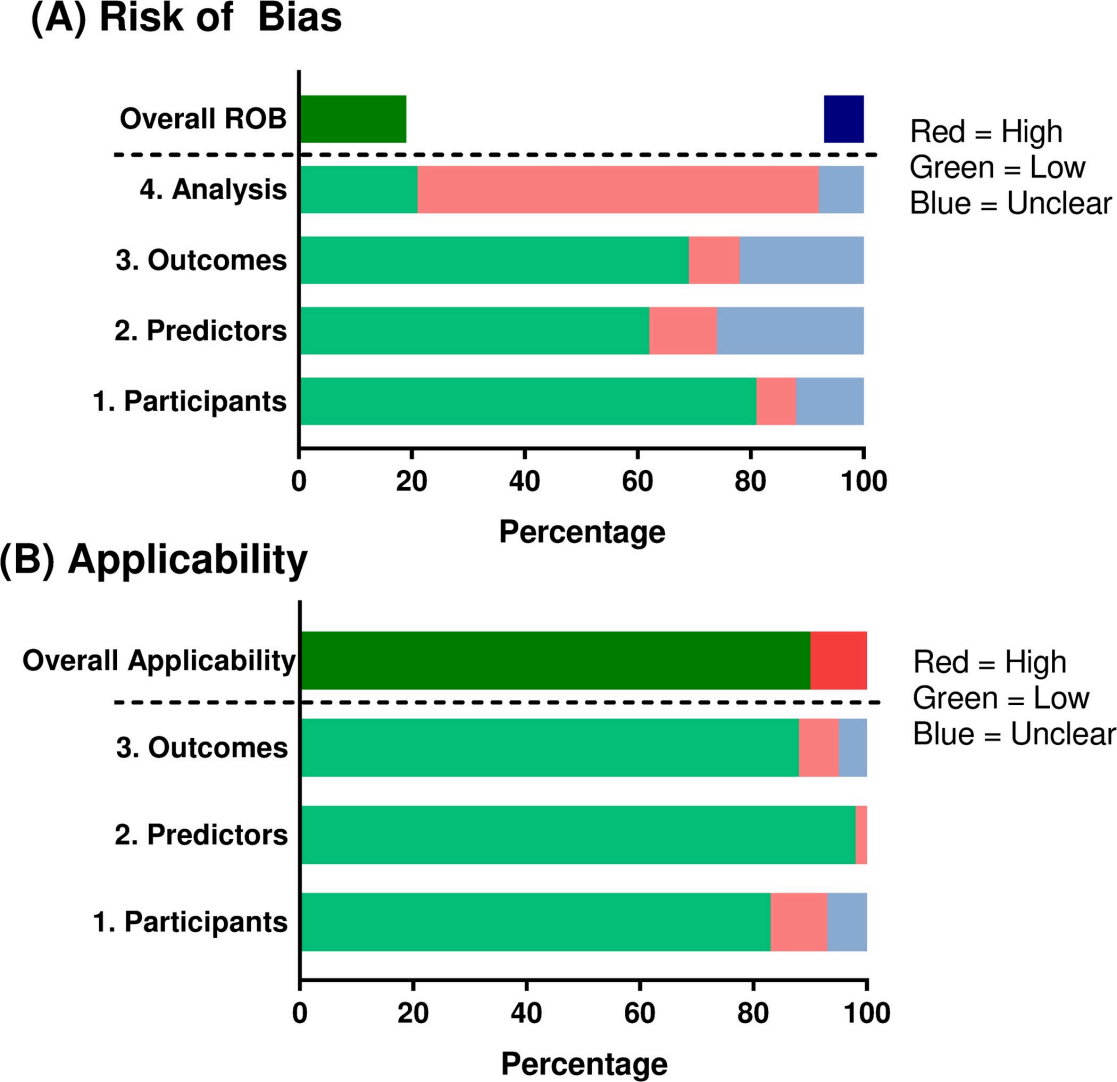
Risk of bias assessment according to the Prediction model Risk Of Bias ASsessment Tool (PROBAST) [16]. ROB, risk of bias.

In total, 58 distinct models were identified from the 40 publications. By applying our assessment of PROBAST [32, 35], 11 models (19%) were classified as overall low ROB, 4 (7%) as overall unclear, and the majority (43 [74%]) as overall high ROB (Fig 3). Of the 11 models considered overall low ROB, (co)primary outcomes across the 4 categories were modeled. Although 11 models (from 7 studies) were rated as overall low ROB according to our assessment of PROBAST, only 3 models had “Yes [Y]” or “Partial Yes [PY]” in all domains of PROBAST. The other 8 models were considered overall low ROB according to PROBAST, despite being rated “Unclear” within at least one Domain (1–3). Of the overall low ROB models, 4 also had an “N” in 1 category of Domain 4. For example, Cubbon et al. [32] had an “N” in Domain 4.1 (“*Were there a reasonable number of participants with the outcome*?”), due to the events per variable (i.e., subjects/variables) being <10 [16]; however, as this model assessing HF rehospitalization was externally validated, it was considered overall low ROB according to Moons et al. [16]. Eapen et al. [35] developed 3 models, and split their data set 70%/30% leading to an “N” in Domain 4.3 (“*Were all enrolled participants included in the analysis*?”). The authors used the 30% split to validate 70% of their data, and as the models were also calibrated, these models were considered overall low ROB according to our interpretation of Moons et al. [16].

Most of the models considered as overall high ROB had a “Y” in multiple signaling questions, but in particular for Domain 4, which assessed model design and validation (S2 Appendix). Zai et al. [60] was rated high ROB on all 4 domains, mainly through lack of reporting. Of the 43 models rated overall high ROB, 32 were ranked “Low” or “Unclear” on the first 3 PROBAST domains assessing participants, predictors, and outcomes, but were classified overall high ROB due to “N” or “PN” in ≥1 aspect of Domain 4 (S2 Appendix). Most often, for these 32 models and overall, an “N” was included in Domain 4.8, which assessed model overfitting and optimism, particularly involving internal validation [16, 17]. For example, Ford et al. assessed 4 co-primary outcomes using 4 models [37]. Ford et al. [37] had an “N” in Domain 4.8 as the models were not reported as being internally validated, and a “PY” in Domains 4.2 and 4.9, as information was reported in the appendix only. As the study did not report information on if the models were externally validated, the models were rated overall high ROB.

When the 58 models were assessed according to applicability concerns, just 6 models (from 5 studies) were rated with overall “High” applicability concern. The majority (52 models) were considered overall “Low” concern, following assessment of applicability to participants, predictors, and outcomes (S2 Appendix).

## Discussion

Publications on risk prediction models have become more common in recent years, but distinct prediction models frequently exist for the same outcome or target population. As such, healthcare professionals, policy makers, or guideline committees have competing information regarding which prediction models should be used or recommended [61, 62]. To aid these decisions, SLRs of risk prediction models are increasingly demanded and performed [11–15]. In this review of the past 5 years, we identified 40 studies that reported 58 multivariable models for risk prediction in HF. Despite risk prediction models varying widely, a number of common distinct predictor variables were incorporated into these identified models. As CV disorders manifest from multiple pathophysiological pathways, a multivariable approach would likely offer additional incremental value beyond the use of single predictors.

In total, 33 of the 40 studies retained >1 candidate variables in the initial assessment, and we identified 12 most commonly used variables, as incorporated in more than 5 studies. For example, age and male sex were frequently incorporated into the base model, in-line with them being key risk factors for onset and survival in HF [2, 63]. Although we identified some commonality in predictors, 105 distinct predictors were identified. This highlights real complexity in HF as a condition, but also the interrelated pathological mechanisms that are considered important for predicting risk, and in part highlights some of the confusion around selecting the most appropriate risk prediction models by professionals [10, 14, 61]. Two publications [46, 55] reported the use of machine learning for predicting risk. Both of these studies incorporated an extensive number of candidate variables for model selection (n = 4205 [55] and n = 110 [46]). Machine learning has shown some promise for improving the accuracy of risk prediction, aiming to increase the number of patients identified who could benefit from preventive treatment, while avoiding unnecessary treatment of others [64]. Contradictorily, an analysis of 71 studies suggested that machine learning had no superiority over logistic regression techniques for predicting risk, although comparison of studies was hindered by methodological reporting [65]. Whether such automated processes can markedly augment predictive performance in the HF setting remains unclear and requires further investigation to define a role in evaluating risk prediction.

Of the multivariable models identified, several models provided C-statistics according to a base model in an effort to determine the incremental value when adding the retained candidate variables into the final model. These studies highlight the steps taken to improve discriminatory ability, the range of variables retained in different risk prediction models, and how these seem dependent on HF outcomes and population under study. There was no particular evidence to suggest that differences in sample size, data source, or HF type significantly affected the discriminatory ability of the models to predict HF outcomes, or clear commonality in the variables retained within the final model. However, it is unlikely that one prediction model will suit all types of HF, and risk should be dependent on level of preserved EF [61]. Ensuring that models properly evaluate both calibration and discrimination is a domain on PROBAST (Domain 4.7), and 14 models did not include sufficient level of information on this domain by our application of the tool. The majority of retrieved studies relied on AUC-ROC / C-statistic to define discriminatory value, and newer approaches were not widely adopted [66, 67]. The C-statistic could naively eliminate established risk factors from CV risk prediction scores [68]. However, it remained a challenge to interpret the distribution of those findings particularly as some studies utilized category-dependent NRIs, whereas others employed a category-free NRI technique. These techniques go beyond conventional discrimination methods by facilitating risk reclassification of patients. Measures such as the NRI have their own limitations, for example the NRI is often heavily influenced by the choice of cutoff points used, as well as the inclusion of unnecessary predictors, and generally requires well-calibrated prediction models for these metrics to be clinically meaningful [66, 67]. The concept of risk reclassification has caused much discussion in the literature, with novel decision–analytic measures being proposed [69]. However, as novel risk factors are discovered, sole reliance on the C-statistic to evaluate discriminatory ability of risk predictors has been suggested as ill-advised [67]. A limited number of studies included a reliable approach to evaluate model performance, and less than half evaluated goodness-of-fit by calibration methods. As such, there is clear room for improving the design of risk prediction models away from reliance on the C-statistic, in parallel with research into improving model performance, ensuring validity and enhancing generalizability.

Given the wide variety in models identified, the PROBAST assessment was applied to give further insight into model design and application. Through our application of PROBAST, 11 models (from 7 studies [21, 26, 32, 35, 53, 57, 59]) were suitably designed and published in a way that suggests the model did not introduce bias into the assessment, highlighting that 47 were not sufficiently described. Some lacking areas that arose from analyzing the prediction models included reporting on methods of calibration and discrimination, validation, and the key issue of how missing data were handled using imputation or other techniques.

A lack of full reporting on aspects of validation or overfitting was the domain on which most studies “failed” (Domain 4.8) according to our application of PROBAST. For example, only 26/58 models included sufficient information to confirm studies were internally validated (“Y” on Domain 4.8). The model by Ahmad et al. [22], although sufficient information across aspects of PROBAST was reported, did not report information on internal or external validation, and therefore was rated overall high ROB, despite being rated low ROB on the first 3 domains of PROBAST, covering participants, predictors, and outcomes. The authors even noted that they did not carry out any method of internal validation [22]. Our observations highlight the need for regular assessments of internal validation and goodness-of-fit, but also the wider adoption of methods of external validation. Importantly, external validation requires measures of both discrimination and calibration in another cohort, and only 8 studies reported information on attempting to use an external model cohort for comparison. Although applicability concerns were low, the PROBAST ROB observations suggested models were generally prone to bias. Introduction of bias could lead to the wrong patients being identified and treated, and ultimately costly mistakes within a healthcare system, if the model was widely used [16, 17]. The risk that patients will be inappropriately treated could partly explain why models are not being confidently used as an aid to HF patient management [61, 62], alongside other concerns discussed in more detail below.

Despite 40 new publications on predicting risk in HF being published within past 5 years, there is little evidence to suggest that any of these 58 models has been adopted by clinicians or healthcare institutions, and no international or local guidance recommending one risk prediction model over another. Indeed, <1% of patients in a European registry received any form of prognostic evaluation [10]. Although many reasons contribute to the limited uptake, poor performance of short-term assessments in guiding decision-making may have contributed [11]. Based on a single-variable model, the GUIDE-IT trial demonstrated that NT-proBNP-guided therapy was not more effective than usual care for improving outcomes in high-risk patients with HF and reduced EF [70]. With such studies, clinicians may therefore see little need to change patient management by risk prediction, seeing all patients as high risk. If risk assessments are to be useful at the bedside, providers need pragmatic models that rely upon easily accessible variables to stratify patients. Given these diverse needs and conflicting evidence of value, further research is required to develop tools, or moreover automated techniques that can provide clinical guidance for risk estimation, in primary care and high-risk or secondary prevention settings [62]. Beyond these concerns, our study highlights the wide variation in statistical approach, the complexity of certain models, and lack of clear external validation, other important considerations for decision makers when recommending any model for predicting risk or stratifying patients according to future risk. Although statistical concerns may hinder clinicians’ confidence with a risk model, development of an app or similar tool to simplify the application of the model for the healthcare provider may also negate the need to fully understand the statistical approach. Clear step-by-step guidance toward the correct patient population would be needed, in an app-type approach.

In order to endorse a risk prediction model within a suitable patient group, decision makers would need to ensure the model is generalizable, as one model may suit given patient groups better than another [61]. Only 13% of identified studies stratified patients by HF type, despite evidence that suggests different models should be used depending on level of preserved EF [61]. In addition, considering patients’ “frailty index” or other functional parameters, such as the 6-min walking distance, within the prognostic modelling of HF may provide a more multidimensional picture of the patient’s risk [71–73]. Further research is needed, however, to ensure the validity of such measures. Patient frailty, for example, can be difficult to interpret [73] and requires additional functional parameters (such as mental, nutritional, or social components) to provide a reasonably accurate definition of “frailty” [72]. However, recent research demonstrated that a frailty index can predict mortality, disability, and hospitalization rates in patients with HF, discriminating from patients without HF [74]. Configuration and use of functional parameters is something that may become more important along with the development of generalizable risk prediction models, but they are still being validated and debated [71, 74]. Further exploration and understanding of automated processes [46, 55, 64] is also needed to help researchers and clinicians gain better insight into the risks and uncertainties involved in the management of different types of HF patient. Collectively, future risk prediction models may involve different measures of function, classification, or clinical usefulness, to give additional insight on the prediction, which extends beyond traditional measures of calibration and discrimination [69].

Some limitations need to be considered when interpreting our observations. We selected a study window of 5 years to ensure we reflect up-to-date knowledge and treatment practices given that HF is a dynamic condition, which often has annual treatment recommendations imposed in many countries. However, by limiting the study window to ensure up-to-date treatment practices were reflected, we did not capture risk prediction models that were published prior to the 5-year window, such as MAGGIC (Meta‐Analysis Global Group in Chronic Heart Failure) or the Seattle Heart Failure Model [75, 76], which had informed contemporary clinical guidelines [77]. Previous reviews, such as that carried out by Rahimi et al., 2014, have included discussion and analysis of these earlier HF models in a contemporary context [12]. Our study time window started after this study by Rahimi et al., but the authors also conclude that although models varied widely, they had some variables in common. In addition, we also found that prediction of HF hospitalization was associated with the lowest discrimination, but that other risk predictions had higher performance that may facilitate clinical use [12], suggesting that discrimination for HF hospitalization has not improved with models developed within the past 5 years and that learnings have not been applied. Just falling outside of our study window, Rich et al. evaluated the MAGGIC risk score (first published in 2013 [75]) for predicting morbidity/mortality in 407 HF patients with preserved EF [78] comparing it with the Seattle Heart Failure Model. The authors concluded the MAGGIC risk score is a valid instrument to assess mortality and morbidity of HF patients with preserved EF and with a better calibration for hospitalization outcome than the Seattle HF instrument. Unfortunately, neither risk model has been assessed with PROBAST.

Each risk model differed, depending on the overall aim of the study, target population considered, length of follow-up, health procedures assessed, location of study, and accessibility to study data, to name but a few. To this end, advocating an optimal modeling approach for use in the HF setting is beyond the scope of this review, and we have discussed some of the limitations around differences in methodologies. Time horizon and sample size varied considerably among the studies identified, with few studies providing sufficient information to confirm robustness and generalizability to qualify the prognosis of individual patients. More rigorous reporting guidance would aid more complete reporting, and in turn, more accurate comparison of studies in an SLR. Nevertheless, by highlighting similarities in approach we hope to inform future decision makers to optimize a model for wider use.

It is clear there is a real need to integrate risk prediction models into healthcare management, but this must be carried out with an eye on bias and handling missing data [61]. Only 28% of studies reported on how they handled missing data. Indeed, most studies (20/40 [50%]) included no information [NI] on how missing data were handled, leading to “NI” in Domain 4.4 of PROBAST. This highlights an area in need of significant improvement in data reporting, to ensure outcomes can be properly concluded upon. Our understanding of the retrieved models is expected to be limited to what is reported within the publication, and the PROBAST assessment should be considered in light of this, as a number of domains were “NI” as no information was available. As such, we cannot disregard the possibility that certain model elements of interest (e.g., as documented in technical modeling reports) may have been overlooked by the present review. Furthermore, the PROBAST checklist is based on reviewer decision-making regarding aspects of the model, which in itself introduces a level of professional decision-making into the assessment of each domain. Therefore, analysis of each study as “high” or “low” ROB should be considered accordingly. As such, independent assessors may come to different decisions regarding domains and models. Further application of PROBAST is therefore required before our observations can be interpreted in light of its application.

## Conclusions

We identified 58 risk prediction models for HF, of which 11 (from 7 studies) were sufficiently detailed and validated to be considered overall low ROB according to PROBAST. The risk prediction models differed with regard to patient population analyzed, their statistical approach, and modeling applied, and confirming prognostic utility was challenging due to the majority of models not establishing a base model. A number of distinct predictors were identified in multiple models suggesting commonality in certain key variables when predicting risk in patients with HF. We feel there is room for improvement beyond what is currently offered in the literature as risk prediction tools for HF, particularly by HF type.

## Supporting information

S1 Appendix Literature search strategy. (PDF)

S2 Appendix Risk of bias assessment. (PDF)

S3 Appendix PRISMA checklist. (PDF)
