# Correction: Evaluating risk prediction models for adults with heart failure: A systematic literature review

**DOI:** 10.1371/journal.pone.0235970

**Published:** 2020-07-02

**Authors:** Gian Luca Di Tanna, Heidi Wirtz, Karen L. Burrows, Gary Globe

In Fig 3, the top, red bar for “Overall ROB” does not appear. Please see the correct Fig 3 here.

**Fig 3:**
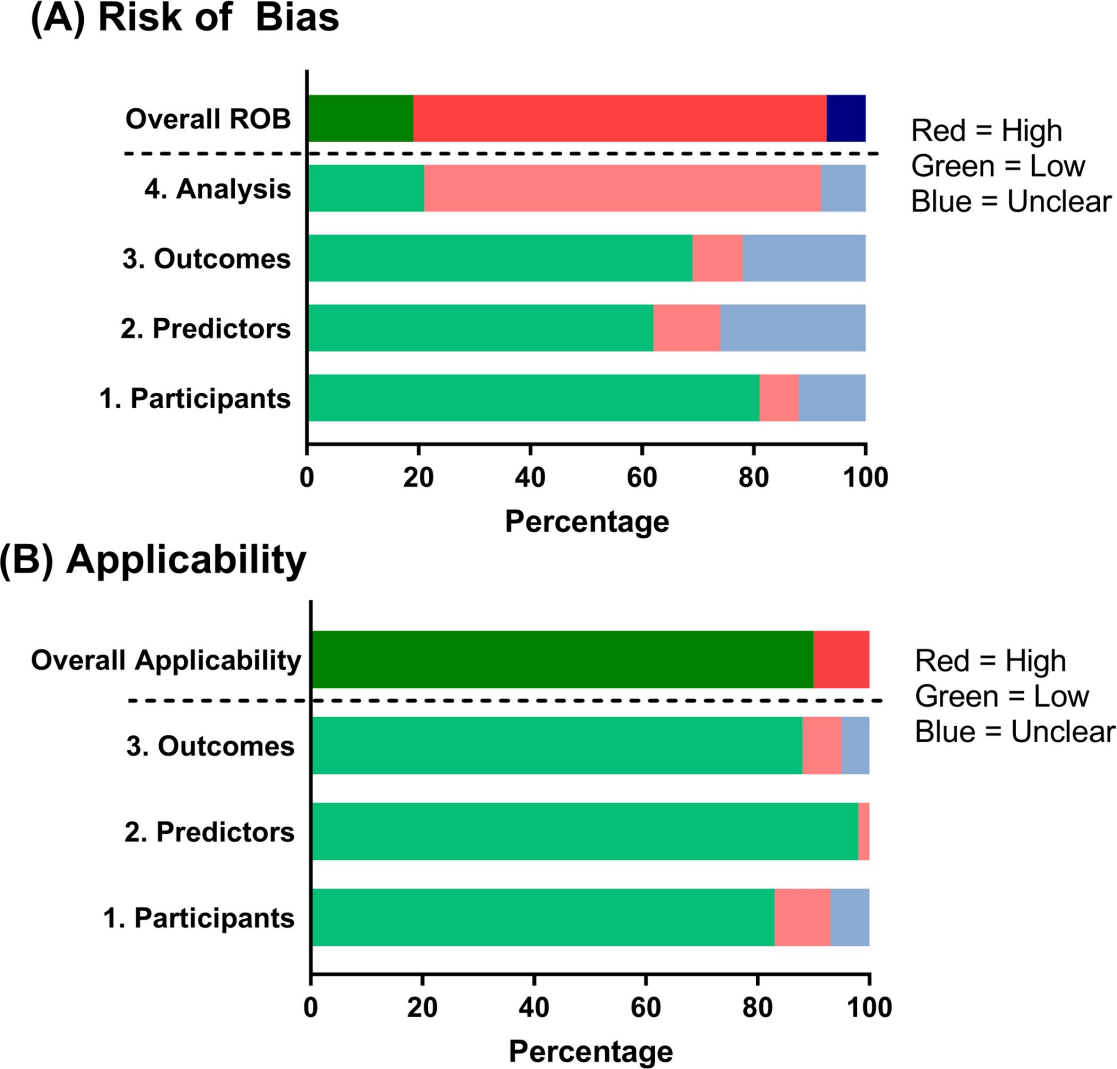
Risk of bias assessment according to the Prediction model Risk Of Bias ASsessment Tool (PROBAST) [16]. ROB, risk of bias.
