# Supplementary material for: Evaluating risk prediction models for adults with heart failure: A systematic literature review
Source: PLoS One. 2020 Jan 15;15(1):e0224135. doi: 10.1371/journal.pone.0224135 (PMC6961879; doi:10.1371/journal.pone.0224135)
Supplement: S1 Appendix — (PDF) [file pone.0224135.s001.pdf]

S1 Appendix. Literature search strategy

Evaluating risk prediction models for adults with heart failure: A systematic literature review

Gian Luca Di Tanna[1] (ORCID ID: 0000-0002-5470-3567), Heidi Wirtz[2], Karen L. Burrows[3], Gary Globe[2*]

[1] Statistics Division, The George Institute for Global Health, Sydney, Australia

[2] Global Health Economics, Amgen Inc., Thousand Oaks, CA, USA

[3] Curo Payer Evidence, Envision Pharma Group, Horsham, UK

*Corresponding author

email: gglobe@amgen.com (GG)

S1A Table. Embase® 1980 to present search.

	Search Term
1	Exp HEART FAILURE/
2	exp congestive heart failure/
3	((heart or cardiac) adj2 failure).ti,ab
4	HF.ti
5	Or/1-4
6	exp hospital readmission/
7	(re admission* or readmission or readmit* or re admit* or re hospitali* or rehospitali*).ti,ab
8	((ambulatory or stable or chronic or out-patient* or outpatient* or post-discharge or urgent or unscheduled* or unplanned or primary care or emergency or office* or out-of-hospital* or critical care or intensive or ICU or ER) adj2 (visit* or appointment* or admission* or call* or stop by* or drop by*

9	OR/6-8
10	*mortality/
11	*death/
12	(mortality OR death*).ti
13	Or/10-12
14	9 or 13
15	5 and 14
16	model*.ti,ab
17	predict*.ti,ab
18	((risk or prognostic) adj2 (factor or score)).ti
19	*risk factor/
20	((Internal or external) adj1 validat*).ti,ab
21	regression analysis/
22	multivariate analysis/
23	biological model/
24	statistical model/
25	Machine learning/
26	Artificial neural network/
27	*algorithm/
28	(multivar* or regression or univar*).ti,ab
29	Net (re)classification.tw
30	Integrated discriminat*.tw
31	Bootstrap*.tw
32	(goodness of fit).tw.
33	(Hosmer Lemeshow).tw
34	OR/16-33
35	Discriminat*.tw
36	(c statistic or area under the curve or AUC).tw
37	35 and 36
38	34 or 37
39	15 and 38
40	(animal not human).sh,hw.

41	39 not 40
42	(editorial or comment or letter or note).pt.
43	41 not 42
44	Limit 43 to English language
45	Limit 44 to dd=20130301-20180529
46	Conference abstract.pt
47	45 not 46

S1B Table. Ovid MEDLINE® in-process & other mon-indexed citations and Ovid MEDLINE® 1946 to present.

	**Search Term**
1	Exp HEART FAILURE/
2	((heart or cardiac) adj2 failure).ti,ab
3	HF.ti
4	Or/1-3
5	exp Patient READMISSION/
6	(re-admission* or readmission or readmit* or re admit* or re- hospitali* or
7	((ambulatory or stable or chronic or out-patient* or outpatient* or post-discharge or urgent or unscheduled* or unplanned or primary care or emergency or office* or out-of-hospital* or critical care or intensive or ICU or ER) adj2 (visit* or
8	OR/5-7
9	*mortality/
10	*death/
11	(mortality OR death*).ti
12	Or/9-11
13	8 or 12
14	4 and 13

15	model*.ti,ab
16	predict*.ti,ab
17	((risk or prognostic) adj2 (factor or score)).ti
18	((Internal or external) adj validat*).ti,ab
19	regression analysis/
20	multivariate analysis/
21	Models, biological/
22	Models, statistical/
23	Artificial Intelligence/ or Machine Learning/
24	"Neural Networks (Computer)"/
25	*algorithm/
26	(Multivar* or regression or Univar*).ti,ab
27	(Net reclassification or net re-classification).tw
28	Integrated discriminat*.tw
29	Bootstrap*.tw
30	(goodness of fit).tw.
31	(Hosmer Lemeshow).tw
32	*risk factors/
33	OR/15-32
34	Discriminat*.tw
35	(c statistic or area under the curve or AUC).tw
36	34 and 35
37	33 or 36
38	14 and 37
39	(animal$ not human$).sh,hw.
40	38 not 39
41	(editorial or comment$ or letter or note).pt.
42	40 not 41
43	Limit 42 to English language
44	Limit 43 to ed=20130301-20180529

S1C Table. Cochrane library search.

	Search Term
#1	MeSH descriptor: [Heart Failure] explode all trees
#2	((heart or cardiac) near/2 failure):ti,ab
#3	#1 or #2
#4	MeSH descriptor: [Patient Readmission] explode all trees
#5	(readmission* or re-admission* or rehospitalisation* or re-hospitalisation* or rehospitalization* or re-hospitalization* or re-admit or readmit):ti,ab
#6	((ambulatory or stable or chronic or out-patient* or outpatient* or post-discharge or urgent or unscheduled or unplanned or primary care or emergency* or office* or out-of-hospital* or critical care or intensive or ICU or ER) near/3 (visit* or appointment* or admission* or call* or stop by* or drop by* or unit* or room* or department* or readmit or re-admit or re-admission or readmission or hospitali*)):ti,ab
#7	#4 or #5 or #6
#8	MeSH descriptor: [Mortality] explode all trees
#9	MeSH descriptor: [Death] explode all trees
#10	(mortality or death or deaths):ti
#11	#8 or #9 or #10
#12	#7 or #11
#13	#3 and #12
#14	model*:ti,ab
#15	predict*:ti,ab
#16	((risk or prognostic) near/2 (factor or score)):ti,ab
#17	((Internal or external) near/1 validat*):ti,ab
#18	MeSH descriptor: [Regression Analysis] this term only
#19	MeSH descriptor: [Multivariate Analysis] this term only
#20	MeSH descriptor: [Models, Biological] this term only
#21	MeSH descriptor: [Models, Statistical] this term only

#22	MeSH descriptor: [Machine Learning] this term only
#23	MeSH descriptor: [Neural Networks (Computer)] explode all trees
#24	MeSH descriptor: [Algorithms] this term only
#25	(multivar* or regression or univar*):ti,ab
#26	(Net reclassification or net re-classification)
