# Supplementary material for: Evaluating risk prediction models for adults with heart failure: A systematic literature review
Source: PLoS One. 2020 Jan 15;15(1):e0224135. doi: 10.1371/journal.pone.0224135 (PMC6961879; doi:10.1371/journal.pone.0224135)
Supplement: S2 Appendix — (PDF) [file pone.0224135.s002.pdf]

S2 Appendix: Supplementary table

Evaluating risk prediction models for adults with heart failure: A systematic literature review

Gian Luca Di Tanna[1] (ORCID ID: 0000-0002-5470-3567), Heidi Wirtz[2], Karen L. Burrows[3], Gary Globe[2*]

[1] Statistics Division, The George Institute for Global Health, Sydney, Australia

[2] Global Health Economics, Amgen Inc., Thousand Oaks, CA, USA

[3] Curo Payer Evidence, Envision Pharma Group, Horsham, UK

*Corresponding author

email: gglobe@amgen.com (GG)

S2 Table: Risk of bias assessment according to the Prediction model Risk Of Bias ASsessment Tool (PROBAST).

Study	Domain 1: Participant			Domain 2: Predictors				Domain 3: Outcome							Domain 4: Analysis*										Overall	
	1.1	1.2	ROB	2.1	2.2	2.3	ROB	3.1	3.2	3.3	3.4	3.5	3.6	ROB	4.1	4.2	4.3	4.4	4.5	4.6	4.7	4.8	4.9	ROB	Applica-bility	ROB
**Adabag S (2014)** [1]	Y	PY	LOW	PN	PY	Y	UC	Y	Y	Y	Y	Y	Y	LOW	Y	PY	NI	NI	PY	Y	Y	PN	PY	LOW	LOW	LOW
**Ahmad T (2014)** [2]	Y	NI	UC	Y	Y	Y	LOW	Y	Y	Y	Y	PY	Y	LOW	Y	PY	PY	Y	Y	Y	Y	N	NI	HIGH	LOW	HIGH
**Alvarez-Garcia J (2015)** [3]	Y	Y	LOW	Y	Y	Y	LOW	Y	Y	Y	Y	NI	Y	UC	Y	Y	NI	PY	N	NI	Y	Y	PN	HIGH	LOW	HIGH
**Barlera S (2013)** [4]	Y	NI	UC	NI	Y	Y	UC	Y	Y	Y	Y	PY	Y	LOW	Y	Y	Y	Y	Y	Y	Y	Y	Y	LOW	LOW	UC
**Behnes M (2016)**† [5]	Y	Y	LOW	Y	Y	Y	LOW	PY	Y	Y	Y	PY	Y	LOW	PN	Y	Y	NI	N	N	Y	N	NI	HIGH	LOW	HIGH
**Betihavas V (2015)** [6]	Y	Y	LOW	Y	Y	Y	LOW	Y	Y	Y	Y	Y	Y	LOW	Y	Y	Y	Y	Y	Y	Y	Y	Y	LOW	LOW	LOW
**Bhandari SS (2016)**‡ [7]	Y	Y	LOW	Y	Y	Y	LOW	PY	Y	Y	Y	PY	Y	LOW	Y	NI	Y	NI	N	NI	Y	N	NI	HIGH	LOW	HIGH

**Bjurman C (2015)†** [8]	Y	PY	LOW	Y	Y	Y	LOW	Y	Y	Y	Y	PY	Y	LOW	N	NI	Y	Y	N	NI	N	N	NI	HIGH	LOW	HIGH
**Cabassi A (2013)** [9]	Y	Y	LOW	Y	Y	Y	LOW	NI	NI	NI	NI	NI	PY	UC	N	NI	N	N	PY	Y	Y	Y	NI	HIGH	LOW	HIGH
**Carluccio E (2013)** [10]	Y	Y	LOW	PY	Y	Y	LOW	Y	Y	Y	Y	PY	Y	LOW	Y	Y	Y	Y	Y	Y	PN	N	N	HIGH	LOW	HIGH
**Carrasco - Sanchez FJ (2014)** [11]	Y	Y	LOW	Y	Y	Y	LOW	Y	Y	Y	Y	PY	Y	LOW	Y	N	N	N	N	PN	N	N	NI	HIGH	HIGH	HIGH
**Cubbon RM (2014)** [12]	Y	PY	LOW	Y	Y	Y	LOW	Y	Y	Y	Y	PY	Y	LOW	N	PY	NI	NI	PY	PY	Y	Y	Y	LOW	LOW	LOW
**Demissei BG (2016)†** [13]	Y	Y	LOW	Y	Y	Y	LOW	NI	Y	Y	Y	NI	Y	UC	Y	Y	N	Y	N	Y	Y	N	Y	HIGH	LOW	HIGH
**Demissei BG (2017)†** [14]	Y	N	HIGH	Y	Y	Y	LOW	PY	Y	Y	Y	PY	PY	LOW	Y	N	Y	NI	PY	NI	NI	Y	Y	HIGH	HIGH	HIGH
**Eapen ZJ (2013)‡** [15]	PY	PY	LOW	NI	Y	Y	UC	Y	Y	Y	Y	PY	Y	LOW	Y	Y	N	Y	NI	Y	Y	Y	PY	LOW	LOW	LOW
**Fleming LM (2014)** [16]	PY	NI	UC	PY	N	Y	HIGH	PY	Y	Y	Y	PN	NI	UC	PY	Y	Y	NI	NI	Y	Y	Y	Y	UC	LOW	UC
**Ford I (2015)§** [17]	Y	PY	LOW	NI	NI	Y	UC	Y	PY	Y	Y	PY	Y	LOW	Y	PY	Y	NI	Y	Y	Y	N	PY	HIGH	LOW	HIGH
**Formiga F (2017)** [18]	Y	Y	LOW	Y	Y	Y	LOW	Y	Y	Y	Y	NI	Y	UC	Y	Y	Y	N	Y	NI	N	N	NI	HIGH	LOW	HIGH
**Freudenberger RS (2016)** [19]	Y	Y	LOW	PN	Y	Y	HIGH	NI	NI	NI	NI	NI	Y	HIGH	Y	N	NI	NI	Y	Y	Y	Y	N	HIGH	LOW	HIGH
**Frigola-Capell E (2013)** [20]	Y	Y	LOW	Y	Y	Y	LOW	Y	Y	Y	Y	Y	Y	LOW	Y	PN	Y	PY	Y	PY	Y	N	PY	HIGH	LOW	HIGH
**Hummel SL (2013)** [21]	NI	NI	UC	NI	PY	Y	UC	PN	N	Y	Y	PY	NI	HIGH	Y	N	N	N	Y	Y	Y	N	PY	HIGH	LOW	HIGH
**Huynh QL (2016)†** [22]	Y	Y	LOW	Y	Y	Y	LOW	Y	Y	Y	Y	PY	Y	LOW	PY	NI	Y	NI	N	NI	Y	NI	NI	HIGH	LOW	HIGH
**Jackson CE (2016)** [23]	Y	Y	LOW	Y	NI	Y	LOW	Y	Y	Y	Y	PY	Y	LOW	Y	Y	PY	NI	Y	NI	Y	Y	PY	UC	LOW	UC
**Jin M (2017)** [24]	Y	Y	LOW	N	Y	Y	HIGH	Y	Y	Y	Y	PY	Y	LOW	Y	N	PY	NI	N	N	N	N	NI	HIGH	HIGH	HIGH
**Keteyian SJ (2016)** [25]	Y	NI	UC	Y	Y	Y	LOW	Y	Y	Y	Y	PY	Y	LOW	Y	Y	Y	NI	NI	Y	PN	Y	NI	HIGH	HIGH	HIGH
**Krumholz HM (2016)** [26]	Y	Y	LOW	PN	Y	Y	HIGH	Y	Y	Y	Y	PY	Y	LOW	N	Y	Y	Y	Y	Y	Y	Y	Y	HIGH	LOW	HIGH

**Lassus J (2013)** [27]	Y	Y	LOW	PN	PY	Y	LOW	NI	NI	Y	NI	PY	Y	UC	Y	PN	PN	N	PY	NI	PN	NI	NI	HIGH	LOW	HIGH
**Lenzi J (2016)** [28]	Y	Y	LOW	PY	PY	Y	LOW	PY	Y	Y	Y	PY	Y	LOW	PN	NI	Y	Y	N	NI	Y	Y	N	HIGH	LOW	HIGH
**Leong KT (2017)** [29]	Y	Y	LOW	NI	Y	Y	UC	Y	Y	Y	Y	PY	Y	LOW	Y	PN	Y	NI	Y	PY	N	Y	PY	HIGH	LOW	HIGH
**Masson S (2018)§** [30]	Y	Y	LOW	Y	Y	Y	LOW	NI	Y	Y	NI	NI	Y	UC	Y	Y	PY	NI	N	Y	Y	NI	NI	HIGH	LOW	HIGH
**Meijers WC (2015)** [31]	Y	PY	UC	N	NI	Y	HIGH	Y	Y	Y	Y	PY	Y	LOW	N	N	NI	NI	N	Y	Y	Y	PY	HIGH	LOW	HIGH
**Montero-Perez-Barquero M (2015)** [32]	Y	NI	LOW	PN	Y	Y	HIGH	Y	Y	Y	Y	NI	Y	UC	Y	NI	N	PY	NI	NI	Y	Y	PN	HIGH	LOW	HIGH
**Nymo SH (2017)** [33]	Y	Y	LOW	PY	PY	Y	LOW	NI	NI	Y	PY	PY	NI	UC	PY	Y	Y	Y	Y	Y	Y	Y	PY	LOW	LOW	LOW
**Ramirez J (2017)†** [34]	Y	PY	LOW	Y	NI	Y	LOW	Y	Y	Y	Y	Y	Y	LOW	Y	PY	PY	NI	Y	Y	N	N	PY	HIGH	LOW	HIGH
**Shameer K (2017)** [35]	Y	N	HIGH	PY	Y	Y	LOW	N	Y	Y	PN	Y	Y	HIGH	Y	Y	PN	NI	Y	Y	PY	Y	PY	UC	LOW	HIGH
**Sudhakar S (2015)** [36]	Y	PY	LOW	PY	Y	Y	LOW	N	Y	Y	Y	PY	Y	HIGH	Y	NI	Y	NI	Y	NI	Y	NI	PY	UC	LOW	HIGH
**Upshaw JN (2016)‡** [37]	PY	Y	LOW	NI	Y	Y	UC	PY	PY	Y	Y	PY	Y	LOW	Y	Y	Y	PY	Y	Y	Y	Y	PY	LOW	LOW	LOW
**Uszko-Lencer N (2017)** [38]	PY	NI	UC	Y	NI	Y	UC	Y	Y	Y	PY	PY	Y	LOW	Y	PY	NI	NI	Y	Y	Y	Y	NI	UC	LOW	UC
**Vader JM (2016)** [39]	Y	Y	LOW	Y	Y	Y	LOW	Y	Y	Y	Y	Y	Y	LOW	Y	Y	PY	Y	PY	PY	PY	Y	Y	LOW	LOW	LOW
**Zai AH (2013)** [40]	PN	NI	HIGH	N	PY	Y	HIGH	NI	NI	NI	NI	NI	NI	HIGH	Y	NI	PY	NI	NI	NI	NI	N	PY	HIGH	HIGH	HIGH

Studies that are overall low ROB are shaded.

*PROBAST is structured as 4 key domains, and we ensured that it was evaluated based on a final model or the decision would have not occurred had there been multiple models.

Studies that included more than 1 final model are listed once in the table. †study had 2 models; ‡study had 3 models; §study had 4 models. A total of 58 models (from 40 studies) were analyzed.

N, No; NI, No Information; PN, Probably No; PY, Probably Yes; ROB, risk of bias; UC, unclear; Y, Yes.

References

1. Adabag S, Rector TS, Anand IS, McMurray JJ, Zile M, Komajda M, et al. A prediction model for sudden cardiac death in patients with heart failure and preserved ejection fraction. Eur J Heart Fail. 2014;16(11):1175-82. Epub 2014/10/11. doi: 10.1002/ejhf.172. PubMed PMID: 25302657.
2. Ahmad T, Fiuzat M, Neely B, Neely ML, Pencina MJ, Kraus WE, et al. Biomarkers of myocardial stress and fibrosis as predictors of mode of death in patients with chronic heart failure. JACC Heart Fail. 2014;2(3):260-8. Epub 2014/06/24. doi: 10.1016/j.jchf.2013.12.004. PubMed PMID: 24952693; PubMed Central PMCID: PMCPMC4224312.
3. Álvarez-García J, Ferrero-Gregori A, Puig T, Vázquez R, Delgado J, Pascual-Figal D, et al. A simple validated method for predicting the risk of hospitalization for worsening of heart failure in ambulatory patients: the Redin-SCORE. Eur J Heart Fail. 2015;17(8):818-27. Epub 2015/05/27. doi: 10.1002/ejhf.287. PubMed PMID: 26011392; PubMed Central PMCID: PMC5032982.
4. Barlera S, Tavazzi L, Franzosi MG, Marchioli R, Raimondi E, Masson S, et al. Predictors of mortality in 6975 patients with chronic heart failure in the Gruppo Italiano per lo Studio della Streptochinasi nell'Infarto Miocardico-Heart Failure trial: proposal for a nomogram. Circ Heart Fail. 2013;6(1):31-9. Epub 2012/11/16. doi: 10.1161/CIRCHEARTFAILURE.112.967828. PubMed PMID: 23152490.
5. Behnes M, Bertsch T, Weiss C, Ahmad-Nejad P, Akin I, Fastner C, et al. Triple head-to-head comparison of fibrotic biomarkers galectin-3, osteopontin and gremlin-1 for long-term prognosis in suspected and proven acute heart failure patients. Int J Cardiol. 2016;203:398-406. Epub 2015/11/06. doi: 10.1016/j.ijcard.2015.10.127. PubMed PMID: 26539964.
6. Betihavas V, Frost SA, Newton PJ, Macdonald P, Stewart S, Carrington MJ, et al. An Absolute Risk Prediction Model to Determine Unplanned Cardiovascular Readmissions for Adults with Chronic Heart Failure. Heart Lung Circ. 2015;24(11):1068-73. Epub 2015/06/07. doi: 10.1016/j.hlc.2015.04.168. PubMed PMID: 26048319.
7. Bhandari SS, Narayan H, Jones DJ, Suzuki T, Struck J, Bergmann A, et al. Plasma growth hormone is a strong predictor of risk at 1 year in acute heart failure. Eur J Heart Fail. 2016;18(3):281-9. Epub 2015/12/17. doi: 10.1002/ejhf.459. PubMed PMID: 26670643.
8. Bjurman C, Holmstrom A, Petzold M, Hammarsten O, Fu ML. Assessment of a multi-marker risk score for predicting cause-specific mortality at three years in older patients with heart failure and reduced ejection fraction. Cardiol J. 2015;22(1):31-6. Epub 2014/02/15. doi: 10.5603/CJ.a2014.0017. PubMed PMID: 24526512.
9. Cabassi A, de Champlain J, Maggiore U, Parenti E, Coghi P, Vicini V, et al. Prealbumin improves death risk prediction of BNP-added Seattle Heart Failure Model: results from a pilot study in elderly chronic heart failure patients. Int J Cardiol. 2013;168(4):3334-9. Epub 2013/04/30. doi: 10.1016/j.ijcard.2013.04.039. PubMed PMID: 23623341.
10. Carluccio E, Dini FL, Biagioli P, Lauciello R, Simioniuc A, Zuchi C, et al. The 'Echo Heart Failure Score': an echocardiographic risk prediction score of mortality in systolic heart failure. Eur J Heart Fail. 2013;15(8):868-76. Epub 2013/03/21. doi: 10.1093/eurjhf/hft038. PubMed PMID: 23512095.
11. Carrasco-Sanchez FJ, Perez-Calvo JI, Morales-Rull JL, Galisteo-Almeda L, Paez-Rubio I, Baron-Franco B, et al. Heart failure mortality according to acute variations in N-terminal pro B-type natriuretic peptide and cystatin C levels. J Cardiovasc Med (Hagerstown). 2014;15(2):115-21. Epub 2014/02/14. doi: 10.2459/JCM.0b013e3283654bab. PubMed PMID: 24522084.
12. Cubbon RM, Woolston A, Adams B, Gale CP, Gilthorpe MS, Baxter PD, et al. Prospective development and validation of a model to predict heart failure hospitalisation. Heart. 2014;100(12):923-9. Epub 2014/03/22. doi: 10.1136/heartjnl-2013-305294. PubMed PMID: 24647052; PubMed Central PMCID: PMC4033182.
13. Demissei BG, Valente MA, Cleland JG, O'Connor CM, Metra M, Ponikowski P, et al. Optimizing clinical use of biomarkers in high-risk acute heart failure patients. Eur J Heart Fail. 2016;18(3):269-80. Epub 2015/12/05. doi: 10.1002/ejhf.443. PubMed PMID: 26634889.
14. Demissei BG, Postmus D, Cleland JG, O'Connor CM, Metra M, Ponikowski P, et al. Plasma biomarkers to predict or rule out early post-discharge events after hospitalization for acute heart failure. Eur J Heart Fail. 2017;19(6):728-38. Epub 2017/03/03. doi: 10.1002/ejhf.766. PubMed PMID: 28251755.

15. Eapen ZJ, Liang L, Fonarow GC, Heidenreich PA, Curtis LH, Peterson ED, et al. Validated, electronic health record deployable prediction models for assessing patient risk of 30-day rehospitalization and mortality in older heart failure patients. JACC Heart Fail. 2013;1(3):245-51. Epub 2014/03/14. doi: 10.1016/j.jchf.2013.01.008. PubMed PMID: 24621877.
16. Fleming LM, Gavin M, Piatkowski G, Chang JD, Mukamal KJ. Derivation and validation of a 30-day heart failure readmission model. Am J Cardiol. 2014;114(9):1379-82. Epub 2014/09/10. doi: 10.1016/j.amjcard.2014.07.071. PubMed PMID: 25200338.
17. Ford I, Robertson M, Komajda M, Bohm M, Borer JS, Tavazzi L, et al. Top ten risk factors for morbidity and mortality in patients with chronic systolic heart failure and elevated heart rate: The SHIFT Risk Model. Int J Cardiol. 2015;184:163-9. Epub 2015/02/24. doi: 10.1016/j.ijcard.2015.02.001. PubMed PMID: 25703424.
18. Formiga F, Masip J, Chivite D, Corbella X. Applicability of the heart failure Readmission Risk score: A first European study. Int J Cardiol. 2017;236:304-9. Epub 2017/04/15. doi: 10.1016/j.ijcard.2017.02.024. PubMed PMID: 28407978.
19. Freudenberger RS, Cheng B, Mann DL, Thompson JL, Sacco RL, Buchsbaum R, et al. The first prognostic model for stroke and death in patients with systolic heart failure. J Cardiol. 2016;68(2):100-3. Epub 2015/11/10. doi: 10.1016/j.jjcc.2015.09.014. PubMed PMID: 26549533.
20. Frigola-Capell E, Comin-Colet J, Davins-Miralles J, Gich-Saladich I, Wensing M, Verdu-Rotellar JM. Trends and predictors of hospitalization, readmissions and length of stay in ambulatory patients with heart failure. Rev Clin Esp (Barc). 2013;213(1):1-7. Epub 2012/12/26. doi: 10.1016/j.rce.2012.10.006. PubMed PMID: 23266127.
21. Hummel SL, Ghalib HH, Ratz D, Koelling TM. Risk stratification for death and all-cause hospitalization in heart failure clinic outpatients. Am Heart J. 2013;166(5):895-903 e1. Epub 2013/11/02. doi: 10.1016/j.ahj.2013.09.002. PubMed PMID: 24176446; PubMed Central PMCID: PMC3896299.
22. Huynh QL, Negishi K, Blizzard L, Saito M, De Pasquale CG, Hare JL, et al. Mild cognitive impairment predicts death and readmission within 30days of discharge for heart failure. Int J Cardiol. 2016;221:212-7. Epub 2016/07/13. doi: 10.1016/j.ijcard.2016.07.074. PubMed PMID: 27404677.
23. Jackson CE, Haig C, Welsh P, Dalzell JR, Tsorlalis IK, McConnachie A, et al. The incremental prognostic and clinical value of multiple novel biomarkers in heart failure. Eur J Heart Fail. 2016;18(12):1491-8. Epub 2016/04/27. doi: 10.1002/ejhf.543. PubMed PMID: 27114189.
24. Jin M, Wei S, Gao R, Wang K, Xu X, Yao W, et al. Predictors of Long-Term Mortality in Patients With Acute Heart Failure. Int Heart J. 2017;58(3):409-15. Epub 2017/05/13. doi: 10.1536/ihj.16-219. PubMed PMID: 28496020.
25. Keteyian SJ, Patel M, Kraus WE, Brawner CA, McConnell TR, Pina IL, et al. Variables Measured During Cardiopulmonary Exercise Testing as Predictors of Mortality in Chronic Systolic Heart Failure. J Am Coll Cardiol. 2016;67(7):780-9. Epub 2016/02/20. doi: 10.1016/j.jacc.2015.11.050. PubMed PMID: 26892413; PubMed Central PMCID: PMC4761107.
26. Krumholz HM, Chaudhry SI, Spertus JA, Mattera JA, Hodshon B, Herrin J. Do Non-Clinical Factors Improve Prediction of Readmission Risk?: Results From the Tele-HF Study. JACC Heart Fail. 2016;4(1):12-20. Epub 2015/12/15. doi: 10.1016/j.jchf.2015.07.017. PubMed PMID: 26656140; PubMed Central PMCID: PMC5459404.
27. Lassus J, Gayat E, Mueller C, Peacock WF, Spinar J, Harjola VP, et al. Incremental value of biomarkers to clinical variables for mortality prediction in acutely decompensated heart failure: the Multinational Observational Cohort on Acute Heart Failure (MOCA) study. Int J Cardiol. 2013;168(3):2186-94. Epub 2013/03/30. doi: 10.1016/j.ijcard.2013.01.228. PubMed PMID: 23538053.
28. Lenzi J, Avaldi VM, Hernandez-Boussard T, Descovich C, Castaldini I, Urbinati S, et al. Risk-adjustment models for heart failure patients' 30-day mortality and readmission rates: the incremental value of clinical data abstracted from medical charts beyond hospital discharge record. BMC Health Serv Res. 2016;16:473. Epub 2016/09/08. doi: 10.1186/s12913-016-1731-9. PubMed PMID: 27600617; PubMed Central PMCID: PMC5012069.
29. Leong KT, Wong LY, Aung KC, Macdonald M, Cao Y, Lee S, et al. Risk Stratification Model for 30-Day Heart Failure Readmission in a Multiethnic South East Asian Community. Am J Cardiol. 2017;119(9):1428-32. Epub 2017/03/18. doi: 10.1016/j.amjcard.2017.01.026. PubMed PMID: 28302271.

30. Masson S, Batkai S, Beermann J, Bar C, Pfanne A, Thum S, et al. Circulating microRNA-132 levels improve risk prediction for heart failure hospitalization in patients with chronic heart failure. Eur J Heart Fail. 2018;20(1):78-85. Epub 2017/10/14. doi: 10.1002/ejhf.961. PubMed PMID: 29027324.
31. Meijers WC, de Boer RA, van Veldhuisen DJ, Jaarsma T, Hillege HL, Maisel AS, et al. Biomarkers and low risk in heart failure. Data from COACH and TRIUMPH. Eur J Heart Fail. 2015;17(12):1271-82. Epub 2015/10/16. doi: 10.1002/ejhf.407. PubMed PMID: 26466857.
32. Montero-Perez-Barquero M, Manzano L, Formiga F, Roughton M, Coats A, Rodriguez-Artalejo F, et al. Utility of the SENIORS elderly heart failure risk model applied to the RICA registry of acute heart failure. Int J Cardiol. 2015;182:449-53. Epub 2015/01/21. doi: 10.1016/j.ijcard.2014.12.173. PubMed PMID: 25602297.
33. Nymo SH, Aukrust P, Kjekshus J, McMurray JJ, Cleland JG, Wikstrand J, et al. Limited Added Value of Circulating Inflammatory Biomarkers in Chronic Heart Failure. JACC Heart Fail. 2017;5(4):256-64. Epub 2017/04/01. doi: 10.1016/j.jchf.2017.01.008. PubMed PMID: 28359413.
34. Ramirez J, Orini M, Minchole A, Monasterio V, Cygankiewicz I, Bayes de Luna A, et al. Sudden cardiac death and pump failure death prediction in chronic heart failure by combining ECG and clinical markers in an integrated risk model. PLoS One. 2017;12(10):e0186152. Epub 2017/10/12. doi: 10.1371/journal.pone.0186152. PubMed PMID: 29020031; PubMed Central PMCID: PMC5636125.
35. Shameer K, Johnson KW, Yahi A, Miotto R, Li LI, Ricks D, et al. Predictive Modeling of Hospital Readmission Rates Using Electronic Medical Record-Wide Machine Learning: A Case-Study Using Mount Sinai Heart Failure Cohort. Pac Symp Biocomput. 2016;Pacific Symposium on Biocomputing. 22:276-87. PubMed PMID: 617085391.
36. Sudhakar S, Zhang W, Kuo YF, Alghrouz M, Barbajelata A, Sharma G. Validation of the Readmission Risk Score in Heart Failure Patients at a Tertiary Hospital. J Card Fail. 2015;21(11):885-91. Epub 2015/07/26. doi: 10.1016/j.cardfail.2015.07.010. PubMed PMID: 26209002.
37. Upshaw JN, Konstam MA, Klaveren D, Noubary F, Huggins GS, Kent DM. Multistate Model to Predict Heart Failure Hospitalizations and All-Cause Mortality in Outpatients With Heart Failure With Reduced Ejection Fraction: Model Derivation and External Validation. Circ Heart Fail. 2016;9(8). Epub 2016/08/16. doi: 10.1161/CIRCHEARTFAILURE.116.003146. PubMed PMID: 27514751; PubMed Central PMCID: PMC5328587.
38. Uszko-Lencer N, Frankenstein L, Spruit MA, Maeder MT, Gutmann M, Muzzarelli S, et al. Predicting hospitalization and mortality in patients with heart failure: The BARDICHE-index. Int J Cardiol. 2017;227:901-7. Epub 2016/12/05. doi: 10.1016/j.ijcard.2016.11.122. PubMed PMID: 27915084.
39. Vader JM, LaRue SJ, Stevens SR, Mentz RJ, DeVore AD, Lala A, et al. Timing and Causes of Readmission After Acute Heart Failure Hospitalization-Insights From the Heart Failure Network Trials. J Card Fail. 2016;22(11):875-83. PubMed PMID: 27133201.
40. Zai AH, Ronquillo JG, Nieves R, Chueh HC, Kvedar JC, Jethwani K. Assessing hospital readmission risk factors in heart failure patients enrolled in a telemonitoring program. Int J Telemed Appl. 2013;2013:305819. Epub 2013/05/28. doi: 10.1155/2013/305819. PubMed PMID: 23710170; PubMed Central PMCID: PMC3655587.
